# A multiple-alignment based primer design algorithm for genetically highly variable DNA targets

**DOI:** 10.1186/1471-2105-14-255

**Published:** 2013-08-21

**Authors:** Johanna Brodin, Mohan Krishnamoorthy, Gayathri Athreya, Will Fischer, Peter Hraber, Cheryl Gleasner, Lance Green, Bette Korber, Thomas Leitner

**Affiliations:** 1 Theoretical Division, Los Alamos National Laboratory, Los Alamos, NM 87545, USA; 2 Department of Microbiology, Tumor and Cell Biology, Karolinska Institute, SE-17177, Stockholm Sweden; 3 Biological Division, Los Alamos National Laboratory, Los Alamos, NM 87545, USA

**Keywords:** Primer design, DNA sequencing, Amplicon sequencing, Next-generation sequencing, PCR, Primer dimer, Bio-barcodes, Multiplex

## Abstract

**Background:**

Primer design for highly variable DNA sequences is difficult, and experimental success requires attention to many interacting constraints. The advent of next-generation sequencing methods allows the investigation of rare variants otherwise hidden deep in large populations, but requires attention to population diversity and primer localization in relatively conserved regions, in addition to recognized constraints typically considered in primer design.

**Results:**

Design constraints include degenerate sites to maximize population coverage, matching of melting temperatures, optimizing *de novo* sequence length, finding optimal bio-barcodes to allow efficient downstream analyses, and minimizing risk of dimerization. To facilitate primer design addressing these and other constraints, we created a novel computer program (PrimerDesign) that automates this complex procedure. We show its powers and limitations and give examples of successful designs for the analysis of HIV-1 populations.

**Conclusions:**

PrimerDesign is useful for researchers who want to design DNA primers and probes for analyzing highly variable DNA populations. It can be used to design primers for PCR, RT-PCR, Sanger sequencing, next-generation sequencing, and other experimental protocols targeting highly variable DNA samples.

## Background

Proper primer design is essential for projects where PCR amplification and/or DNA sequencing play an important role, and a number of algorithms and design recommendations have been proposed. Depending on the goal of a particular project, criteria for selection of primers (and probes) include simple parameters like the length of primers and products, as well as considerations regarding the information the researcher wants to extract. Because *in silico* design prior to starting a project may prevent later problems in experiments and analyses, many specialized primer design computer programs have been created. They focus on certain aspects of primer design, including multiplexing, degenerate sites, discriminate amplification, nested PCR, SNP protocols, and hybridization analyses such as micro-arrays and *in situ* hybridization, and of course DNA sequencing [1-8]. Partly because design is complicated, even for well-defined targets with little or no genetic variation, it is usually performed in discrete, non-communicating steps. This is unfortunate because limitations in one step may affect another, and similarly a problem in one step could possibly have been circumvented by an alternative in another step. Thus, when design is undertaken in a series of separate steps, it may be difficult or impossible to optimize the overall design. Therefore, there is a need for a method that can optimize primers and probes while considering all design criteria simultaneously.

Deep sequencing, i.e., re-sequencing of a known DNA sample to reveal relative frequencies of individual population variants and to detect rare mutants, has become feasible thanks to next-generation sequencing (NGS). For example, NGS has been used to investigate early immune escape variants in acute HIV-1 infection [9-12], evolution of distinct phenotypic traits such as transitions in HIV co-receptor usage [13], virus genetic variation during drug treatment [14-16], endogenous siRNAs [17], cancer gene variants [18], and genetic variation in gut microbiota [19]. To accurately amplify and sequence such populations, genetic variation needs to be considered in the primer design. Primer design that captures only limited diversity relative to the sample population diversity, and favors certain variants, will bias the outcome. Thus, in addition to the advantages of designing the physical parameters of primers in parallel rather than in serial steps as discussed above, it is also valuable to consider a multiple-alignment representative of the diversity in the sample of interest in the overall design. It is important to locate primers in relatively conserved regions bounding the region of interest, and to design primers that will adequately address the level of variation that cannot be avoided.

Motivated by the scientific goals of analyzing diverse HIV-1 and SIV populations, and the fact that no general primer design software existed that included both the many steps of the design procedure, as well as the rich diversity information from a multiple-sequence alignment, we developed an algorithm that integrates all these components into a comprehensive tool. This tool was based on our experience with 454 deep sequencing, but can also be used for primer design for other NGS technologies such as IonTorrent, Illumina, SOLiD, PacBio, as well as general PCR and traditional Sanger sequencing protocols. We show examples of the use of this algorithm to successfully analyze diverse HIV-1 populations, both within and between infected patients. Note that although these viruses are among the most genetically variable organisms known [20-24], the tool is not restricted to HIV/SIV design; it can be used to design primers and probes to any aligned set of DNA sequences, regardless of diversity level and organism. To make access easy, guarantee that the latest version of the tool is used, and make the program independent of computer platform, the software is available as a web tool at the LANL HIV database.

## Implementation

### Software organization

This software was built using Perl 5 and C programming languages. The web interface was constructed using the Catalyst Model-View-Controller framework (http://www.catalystframework.org) and Moose object-oriented programming in Perl (http://moose.iinteractive.com).

The software was built in modules, most of them written in Perl 5. Modules that are computationally intensive, such as the dimerization risk estimation, were written in C. Other parts, such as the bio-barcode generation and edit distance filtering have been pre-calculated and are stored in a database for quick access during PrimerDesign optimizations. The bio-barcodes were calculated on a parallel computer, using inline C for Perl.

### Alignment data

To illustrate PrimerDesign performance, we used sequence alignments from one of the most variable organisms known, the human immunodeficiency virus type 1 (HIV-1) [9]. The design is based on a pre-existing multiple DNA sequence alignment.

### PCR and 454 sequencing

PCR pre-amplification was performed as previously described [9,11]. These amplifications were performed with adaptor-tag-primer constructs designed using the methods described in this paper. The pre-amplification boosts the template numbers for subsequent 454 analyses while introducing bio-barcodes for downstream bioinformatic analyses and universal adaptors for 454 emulsion PCR and sequencing.

Sequencing reactions were run on a Roche Genome Sequencer FLX system (Roche, Basel, Switzerland) as previously described [9,11]. Briefly, all samples were diluted and pooled. For emulsion PCR, 2.25 ml of the pooled sample was added to a reaction mix containing 450,000-600,000 capture beads per reaction. The dilution corresponded to copies per bead ratios of approximately 1–10, and the yield was proportional to that ratio. The PCR products were retrieved by breaking the emulsion and enriching for beads contain amplified products. Approximately 400,000-750,000 enriched DNA beads from each reaction were deposited into one of 2 regions of a 70 × 75 mm Pico Titer Plate for sequencing.

## Results and discussion

### Multiple-alignment informs primer design

**Figure 1:**
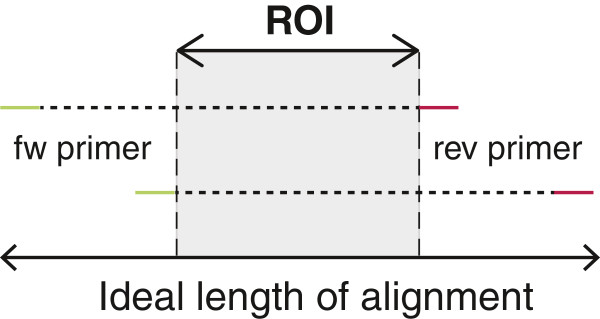
**The region of interest (ROI) defines the *de novo *target DNA sequence.** Ideally a multiple alignment should have ample space for primers on either side of the ROI, considering desired primer lengths and sequence read length (which depends on the sequencing system used).

A high-information multiple sequence alignment covering a region of interest (ROI) must be supplied by the user (Figure 1). The ROI is the DNA region that contains the *de novo* DNA information in the samples to be analyzed. These could be, for example, sites related to drug resistance, CTL epitopes, or other features. It is important that the alignment contains examples of the expected genetic variation in the population to be analyzed, for instance, that sufficient diversity in hyper-variable regions to be representative is included. This will allow the design of primers that both avoid highly variable regions, and that address the diversity found in the more conserved regions that are good candidates for primer localization. To allow localization of the best primer sites, the ROI should be flanked by sequence on either side. Ideally, such flanking sequences should be long enough to allow most sequence reads to extend the full length of the ROI, and maximize the range of possibilities for primer selection. Formally, therefore, flanking sequence length should equal the maximum sequencing read-length minus the ROI-length on either side: e.g., if the ROI is 100 bp and maximum sequencing read length is 500 bp, the alignment should ideally be (500–100) + (500–100) + 100 = 900 bp long. While this may not always be possible, it is helpful as a guideline; at minimum, there must always be enough sequence on either side of the ROI to identify a suitable place for a primer. When studies of within-subject viral diversity are undertaken, having a set of sequences spanning the ROI from the subject is desirable to inform primer design; when sequences will be obtained from multiple subjects, population diversity needs to be considered. While our primer design methods will work using alignments that come from any variable organism, however for HIV we provide many premade alignments that cover different levels of genetic variation or that are made on the fly from database searches at the HIV database site http://www.hiv.lanl.gov.

### User and sequencing method restrictions constrain primer design

When primers are designed for re-sequencing, including NGS-based deep sequencing, the user already has information about the ROI. Based on the alignment, the user specifies the ROI by its alignment-specific start and end coordinates. The user also can place constraints on the primers in terms of minimum and maximum length (10 ≤ *L* ≤ 40 bp), and maximum allowed differences in melting temperatures (*T*_*m*_) in °C to match the primers to experimental conditions and desired *de novo* lengths. Importantly, primers will be designed to include genetic variation down to a user specified detection limit *d*, which, if necessary, will include degenerate nucleotide states at some primer positions. The user can also restrict the design by requiring primer sites to have G/C characters at 3′- and/or 5′-ends.

In addition to user specified constraints, the sequencing method also puts restrictions upon the design process. For instance, if the user specifies 454 Titanium as adaptors, this automatically sets restrictions on the barcode generation as well as possible primer sites, to avoid di-nucleotides of the same state at junctions and the potential for dimerization. Another important method-specific restriction is the total length of the fragment to be sequenced: this is set automatically based on published sequencing lengths for different sequencing methods, but the user can change this by specifying “my adaptor” and a desired *de novo* length.

**Figure 2:**
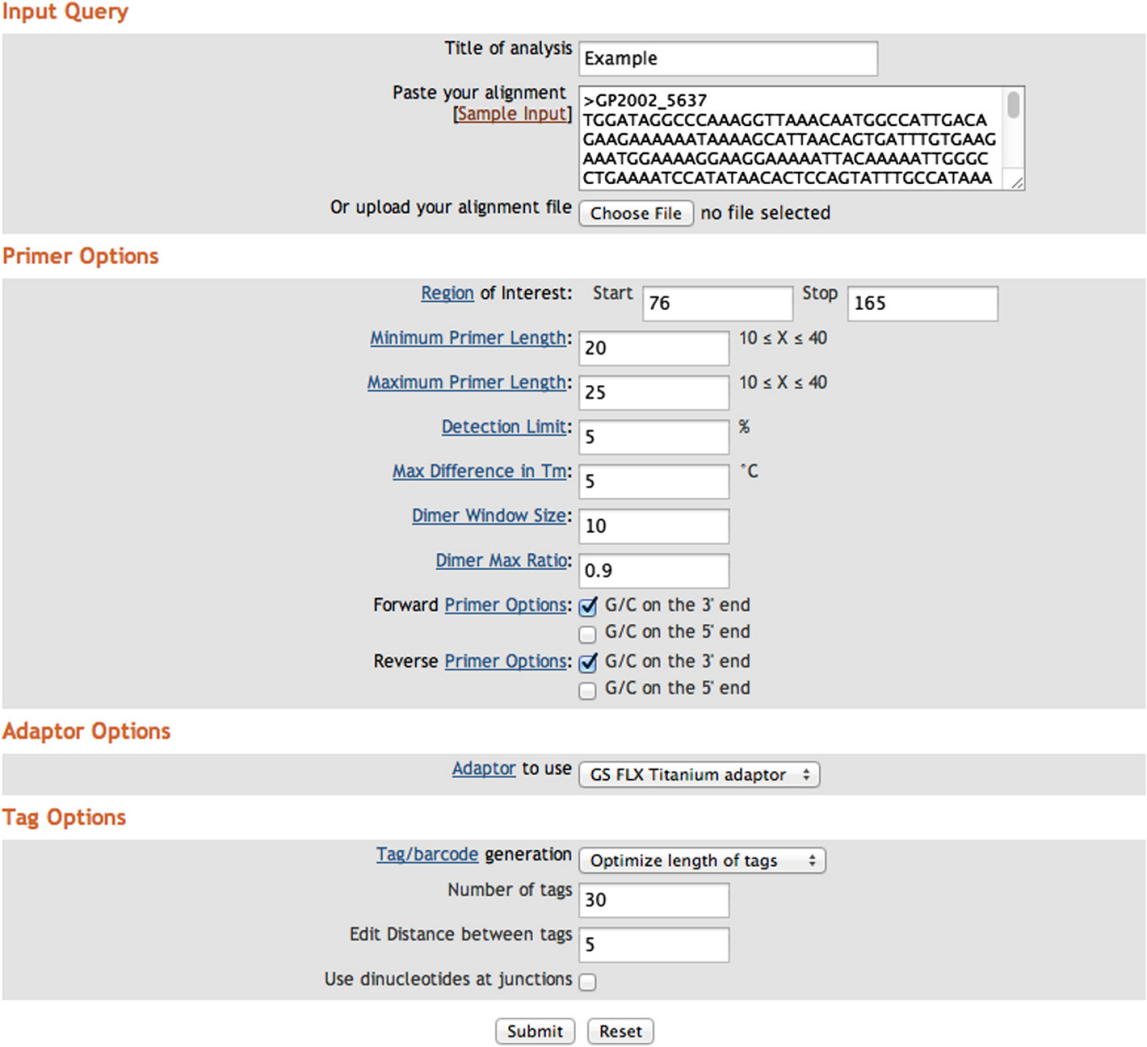
**Input web page for PrimerDesign.** Alignments can be either pasted into the input window or uploaded as a file from the user’s computer. A ‘sample input’ is provided so users can test run the software and familiarize themselves with the effects of different options. This example shows the default primer option values that we provide. All options have a hyperlink to a help page that explains how to use them.

All user constraints are input on a simple web form (Figure 2), and method-specific constraints are automatically enforced based on user information. The resulting primer-constructs are presented in a table format that easily can be transferred to a spreadsheet or database. Up to 5 alternative primer-construct pairs will be presented. In addition, appropriate barcodes will be listed for each primer-construct pair. Such DNA-based barcodes (aka. “tags”) can be used to identify different sample sources, e.g., different patients, longitudinal samples, tissue compartments, etc. This table of primer-construct pairs may become very large, as 1,000's of bar codes can be generated for use with a primer-construct. Importantly, this also makes it possible for a user to design template-specific barcodes [16] and to investigate and predict problems related to barcode misreading or loss of sequence fragments due to barcodes acting as alternative primers.

### Entropy informs about primer locations

In order to find good locations for the primers within the alignment, the algorithm first finds the nucleotide frequencies at each position of the alignment. It then excludes nucleotides whose frequency at any position falls below a desired detection limit *d* (given by the user), and calculates a multi-state consensus character (IUPAC code) representative of the remaining nucleotides at each position. The remaining variability is then estimated by the Shannon entropy for each site [25,26]. Next, the locations of all forward-reverse primer pairs are found around the region of interest (ROI) with primer lengths between minimum and maximum primer length (given by the user) sorted by their entropy scores. Lower entropies are preferred. These potential primer pairs are then filtered by *T*_*m*_ and dimerization risk.

### Estimating *T*_*m*_ for matching to experimental conditions

The primer melting temperature *T*_*m*_ is estimated by the empirical nearest-neighbor model [27,28], assuming a sodium concentration of 0.15 M and primer concentration at 30 μM, and dinucleotide entropies and enthalpies according to published empirical values [27,28]. Because the consensus sequence may contain multistate characters (aka. degenerate sites), each such site is deconvoluted into possible A, C, G, and T characters and all possible primer sequences are generated. The complexity measures how many individual primers composed of only A, C, G, and T characters exist for a given primer (and across the entire alignment). For each of the deconvoluted primers the *T*_*m*_ is calculated, and the average is presented for each potential multistate-containing primer. If the difference in *T*_*m*_ between a forward and a reverse primer is within the maximum allowable limit (given by the user), then that primer-pair is appended to the list of potential constructs and sent to the dimerization risk estimation.

### Bio-barcode tags label sequences

**Figure 3:**
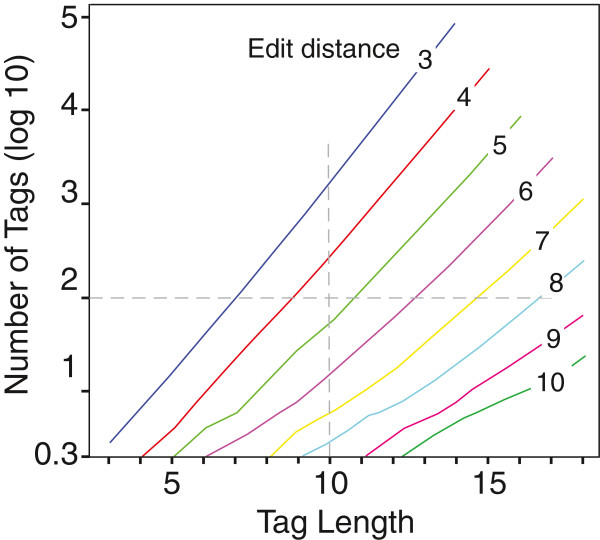
**Current limitations in the number of possible bio-barcode tags for different lengths and edit distances.** These bio-barcodes are stored in a database for fast selection according to user-specified criteria. Two examples (dashed lines) are indicated, representing user requests for 100 bio-barcodes and a length of 10 bp.

The user can optimize the bio-barcode tags based on 1) a desired number of unique tags, 2) a certain length of the tags, or 3) a minimum edit distance. Currently, we have restricted the number of tags to 200,000 and a tag length of 18 nucleotides. The edit distance (Levenshtein distance), i.e., the possible minimum number of “mutations” required to modify one tag into another depends on tag length, can be selected to be up to 10 for long tags. A higher edit distance makes downstream bioinformatic sorting more robust, translating into fewer lost sequences due to ambigious reads, and also fewer misclassifications of barcodes. Figure 3 shows the current limitations on the number of possible tags for different lengths and edit distances. Tag numbers grow exponentially as a function of length, and at lower numbers stochastic limitations due to starting values influence the actual number of possible tags.

To avoid time-consuming generation of barcodes every time the software is run we have constructed prior lists that PrimerDesign accesses during the primer-tag-adaptor design. There are two sets of potential barcodes. The first set contains all tags of lengths between 3 and 18 nucleotides. This list was generated recursively. Thus, for tag length *n*, nucleotides were appended to the tags of length *n*-1. During the generation of the tags, no tags with repeated nucleotides at adjacent sites were allowed as this is known to cause potential misreading in pyrosequencing [29,30]. The second set of tags was derived from the first set where tags were filtered out when the edit distance was less than a user-specified minimum. All sets with minimum edit distances of 3–10 were pre-calculated. In detail, to effectively generate the second set the algorithm checks for the longest common substring of two tags. If the difference between the tag length and the common-substring length was less than the specified edit distance, then the Levenshtein distance between the two tags must also be less than the edit distance. Because the lists are recursive, this procedure led to a great reduction of the number of calculations for longer tag lengths.

### Automated dimerization risk filters design constructs

**Figure 4:**
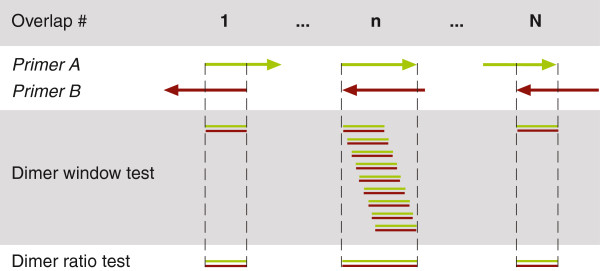
**Prediction of dimerization risk.** The dimerization risk is assessed by virtually sliding one primer (A) across another (B), reversed. Each potential overlap (1…n…N) is examined by i) a sliding window that tests if the two primers have an exact match within the window (user-specified parameter), and ii) a calculation of whether the fraction of matches in the overlap exceeds a certain ratio (user-specified). If either test indicates a potential dimer, the set is discarded. Primer-primer tests occur for all possible homo- and hetero-dimers.

Because primer-dimers may cause serious problems in experiments, we search for potential dimerization risk between all primer constructs (primer-tag-adaptor oligomers) that will be in the same reaction. Both potential homo-dimer (e.g., hairpins) and hetero-dimer structures are investigated. This algorithmic step can become very computationally intensive, especially if large numbers of tags and/or a high degree of complexity are included in the design. Dimerization risk is evaluated in two ways (Figure 4): 1) in a user-defined window moved along all potential primer-primer interactions, and 2) as a user-specified fraction of bonds in an interaction. To reduce the software running time we have removed redundant calculations by hierarchically dividing and testing the different parts of the primer-tag-adaptor constructs. Specifically, the first step is to check for dimers amongst only the adaptors, since all constructs in one reaction have the same adaptors. The second step involves estimating dimer risk in the additional adaptor-primer parts since each primer set will have the same forward-reverse primers. Finally, the third step involves the remaining adaptor-primer-tag parts, as each construct in each primer set will have a different tag. This allows estimating the dimerization risk early, thus reducing the number of calculations and potentially long algorithmic loops that otherwise would slow down the design process. Potential primer-dimers detected during the design are logged in a file that can be downloaded for quality control analyses.

Hence, all unique primer constructs are checked against each other, and if an interaction shows more than the user-specified number or ratio of matches, then that set is discarded. The default values (window = 10; ratio = 0.9) are conservative values based on recently published estimates of dimerization risk [31].

### PrimerDesign suggests primer pairs

**Figure 5:**
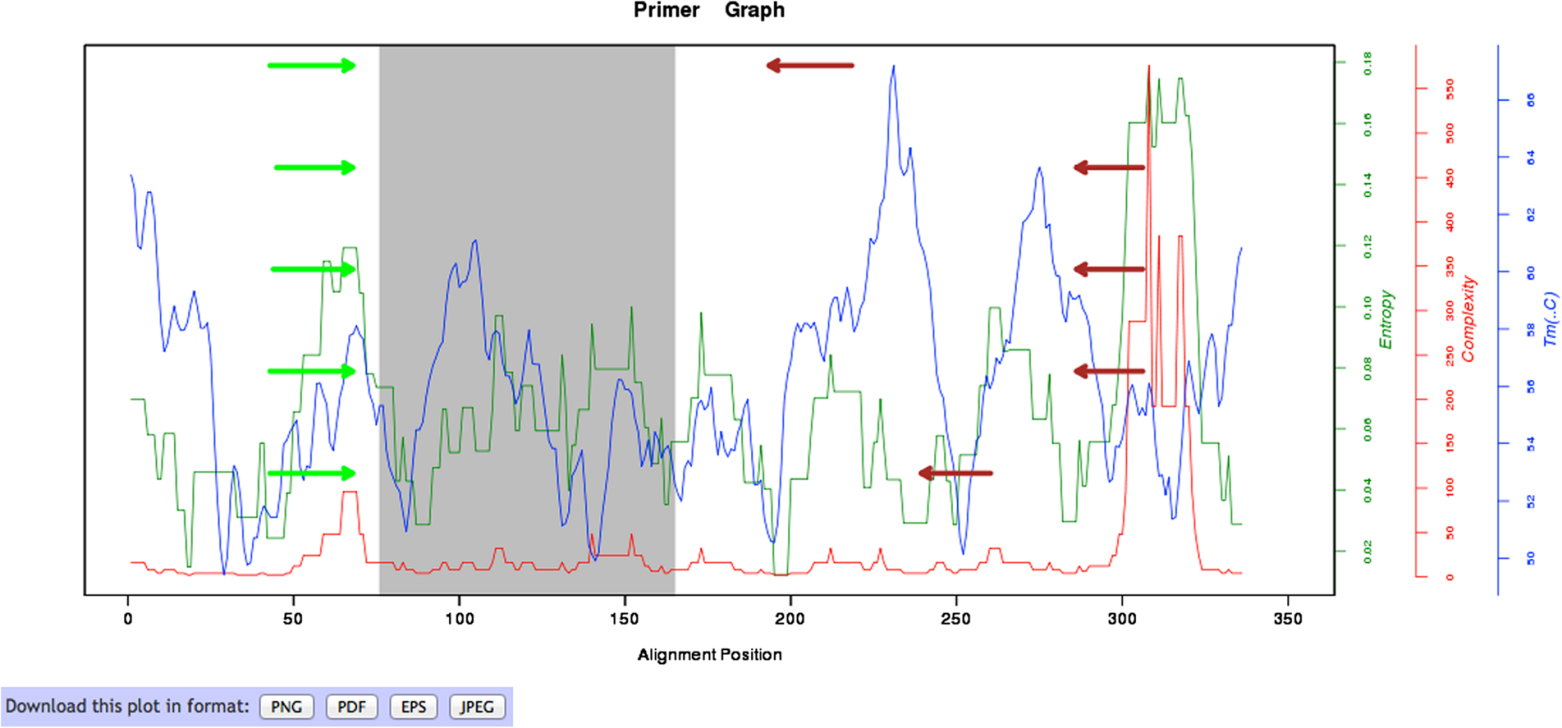
**Example output web page from PrimerDesign.** The output includes a list of the design parameters specified by the user, a graphical illustration showing primer locations (arrows), the ROI (in grey), and curves for entropy, complexity and T_m_, followed by a table of the primer constructs. The output also includes buttons to download the graph in different formats, the primer constructs in tab-delimited format, and the dimerization risk-testing log. This figure shows only the primer graph component, because the table may be very long (depending mainly on how many bio-barcodes were requested).

Based on user specifications, minimization of sequence entropy and complexity, *T*_*m*_-matching, and checking for potential dimerization risks, the five best primer pair constructs are identified graphically in a plot that corresponds to the user-provided alignment on which the design was based (Figure 5). The ROI is marked (in grey) and the five best forward and reverse primer pairs are indicated on either side as arrows. The graph also shows the Shannon entropy (in this example for a hypothetical primer 20 bp long), the complexity as defined by the number of degenerate sites (informed by *d*), and the estimated mean *T*_*m*_. These curves allow the user to interact with the design, e.g., to identify primer sites with less complexity that would be created if the restriction of G/C in the 3′-end were relaxed. If desired, the user can go back and reevaluate the original design. For instance, Illumina’s error rate rises along the length of its reads; therefore, it may not be desirable to place the region of interest at the end of the sequencing read, which experiences the highest error rate. Of course, this can be controlled by the user, by simply reducing the expected read length, therefore placing the primers closer to the ROI.

In addition to the graphical output, we also provide a simple table with the primer constructs and *T*_*m*_, which can be used for primer synthesis and experimental setup, and a log file with additional quality control and iteration data.

The execution speed of the program depends on a number of factors, particularly the size of the input multiple alignment, the level of genetic variation, and the number of barcodes requested. The level of genetic variation affects several steps; as the variability increases, the number of degenerate sites may also increase, which adds to 1) the complexity and the number of primers to average *T*_*m*_ over, and 2) the number of potential primer-dimers. The level of complexity to consider is adjusted by the user-specified detection limit *d*. It is noteworthy in this context that adjusting target *T*_*m*_, and the allowable matching intervals can make primers designed with less complexity/lower detection limit still be sufficiently promiscuous to amplify all genetic variants in the target population. At the web site we provide a “sample input”; this imports an alignment and several parameter values, with no adaptors, *de novo* length of 200 bp, and no barcodes; it finds optimal primers in about 25 seconds, depending on server load and internet speed. When calculations take long times, i.e., when web browsers may time out before the design is completed, we provide an email notification option. This sends an email to the user with a link to the results.

### Primers used in successful 454 sequencing of HIV-1 populations

This PrimerDesign algorithm was used to design primers to investigate CD8 T-cell driven HIV-1 immune escape over time, starting with acute infection samples from 3 human patients [9]. We were also interested in using 454 pyrosequencing to better estimate the number of HIV-1 strains that established the primary infection in these subjects [9]. The *env* V3 region is a highly variable region of the HIV-1 genome, and was therefore, as in a previous HIV-1 deep-sequencing study of long-term infections [14], sequenced as a reference region in all study subjects to examine non-T-cell-selected diversification. Thus for each patient the ROIs were chosen to encompass the *env* V3 region, along with additional regions spanning one or two verified CD8+ T cell epitope(s) recognized early in infection. These regions were located in *nef* in one subject, *rev* and *tat* in the second, and *env* in the third [9]. We were interested in detecting the emergence of low frequency variants in each patient, and tracking the progression of immune escape over time. In addition to the 3 acutely infected patients, we had access to one sample from a chronically infected donor who was expected to have a very diverse virus population. Based on alignments of previous Sanger sequences from these 4 patients [32], the previous 454 study of chronic patients [14], and additional sequences from the HIV database (http://www.hiv.lanl.gov), we designed 30 unique adaptor-tag-primer constructs: 2 patients × 2 regions (a T-cell epitope region and the V3 variable region) × 4 samples per patient (3 longitudinal samples + 1 control) = 16, plus 1 patient × 3 regions (2 T-cell epitope region and the V3 variable region) × 4 samples per patient (3 longitudinal samples + 1 control) = 12, plus 2 regions for the single time-point for the chronically infected donor. Each of these constructs successfully revealed accumulating genetic variation in the investigated regions over time, and extensive within-epitope diversity. The number of sequences retrieved varied in the patients, but was generally similar between regions within the same patient [9]. Furthermore, the variation found in the HIV-1 sequencing experiments agrees very well with results from Sanger sequencing by single-genome amplification (SGA), with 95% confidence intervals for variant frequency detected by SGA overlapping frequencies from 454, and increased sensitivity to detect rare variants [12].

**Figure 6:**
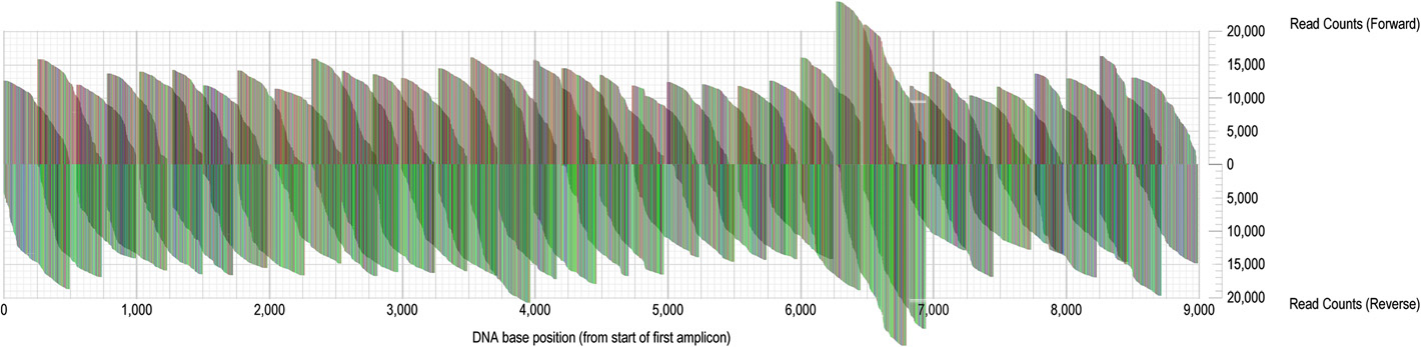
**A visual representation of a portion of a 1.4-gigabase, 4.5-million-sequence nucleotide alignment, derived from overlapping 454 reads covering nearly the complete HIV-1 genome, from 35 individual PCR amplicons from a single patient at 5 time-points during infection.** The “fin-like” structures reflect the length distributions of reads that begin at each primer site (left-to-right for forward reads, above midline; right-to-left for reverse reads, below midline). In the original image, which has been reduced for printing, each pixel corresponds to a single nucleotide (A, red; C, blue; G, black; T, green). Each vertical grid line represents 5,000 sequence reads; each horizontal grid line, 50 base pairs. The overall number of reads across the genome was fairly constant, indicating similar primer design success and data richness for genetic variability assessments.

To further investigate PrimerDesign’s performance in identifying effective primers for revealing genetic variation in different regions of the HIV-1 genome, we designed primer sets to provide overlapping short amplicons across the HIV-1 genome of the HIV-infected subject CH40. We aimed at finding 454 sequencing primers every 250 bp that would work in both directions, using full-length genome sequences obtained from this subject as the an input alignment for primer design [12]. As the expected read-length was about 500 bp, this resulted in a tiled primer design of 30 + 30 primers. Figure 6 shows the aligned 454 sequences covering the entire HIV-1 genome. Critically, most primers generated similar numbers of *de novo* sequences, hence displaying comparable levels of genetic detail across the genome. A few primers around genome positions 6500–7000 gave more data, interestingly in both directions, and none of the primers failed. Because the genetic variation of HIV-1 is highly non-uniform across the genome, this shows that our PrimerDesign algorithm can find good primers regardless of whether a highly variable gene (such as *env*) or a less variable gene (such as *pol*) was the ROI, and that the actual sequencing depth was comparable across the genome.

The generation of primers across the entire HIV-1 genome was done with an in-house semi-automated version of PrimerDesign, but it could be easily done manually using multiple ROIs in separate runs. We may add this as an automatic feature in the future if requested by users, which is customary for LANL HIV database bioinformatic tools. Similarly, we welcome other user requests for further improvements, and we will implement such requests if feasible.

### PrimerDesign compared to other software

The biggest strength of PrimerDesign compared to the majority of previous primer design programs is PrimerDesign’s comprehensive approach. Specifically, PrimerDesign uses a multiple alignment to address deep-sequencing needs of primer localization in relatively conserved regions of highly variable DNA targets, allows the integrated design of bio-barcode tags and adaptors, and allows primer-dimer evaluation based on experimental data. Most primer design software tools base their design on a single sequence (e.g., Primer3 [1]), which could result in selective or biased amplification from a diverse population by inadequate coverage of variants, or inadvertent selection of primers in relatively diverse regions, when conserved regions could be used instead, thereby risking missed coverage due to biological variability in primer regions. Other programs that use multiple alignments (e.g., GeneFisher [5]) lack the ability to simultaneously evaluate primers appropriately, for instance, by matching *T*_*m*_*’*s and checking for dimerization risk. In these comparisons, it is important to point out that many previous primer design programs have focused on specific needs of certain experimental protocols, and therefore comparisons are difficult. Another advantage with PrimerDesign is its automatic and flexible tag generation, which is becoming more important with the increasing usage of multiplexed and next-generation sequencing. Tag generation, together with the possibility of adding DNA adaptors, and testing the entire construct’s dimerization risks, are to our knowledge unique features of PrimerDesign. Primers designed using PrimerDesign could readily be used in conjunction with the Primer ID strategy, where a random sequence tag is introduced so that each template receives a unique primer ID [16]. The goal of the primer ID is to minimize errors in estimating sequence frequencies resulting from initial sequence resampling and amplification biases, and template consensus sequences, and to minimize the impact of recombination during amplification and misincorporation/sequencing errors [16]. The introduction of such tags may introduce some primer issues, as these tags are by definition random, and added at the DNA synthesis stage, and so will not be designed to avoid homopolymer strings, dimerization issues, and some bias may be introduced by the chemistry of synthesis.

## Conclusions

We have created a novel primer design tool, PrimerDesign, which suggests primer pairs according to a comprehensive algorithm and user requirements. The overall software workflow proceeds through inter-connected steps: 1) the target locations for primers are determined, guided by sequence entropy estimates’ and complexity, 2) primer melting temperatures are optimized, 3) bio-barcodes and adaptors are added, and finally 4) dimerization risks are estimated. Each inter-connected step informs the subsequent steps; if previous steps have to be re-optimized, this occurs automatically. Thus, each step considers both user requirements and automatic parameters used within the algorithm.

PrimerDesign was originally designed for HIV studies; however it should be equally useful for designing primers for other biological systems that have high levels of genetic variation. Thus, we have presented a novel computer program for designing primers for highly variable DNA targets. The design takes into account genetic variation, and several user-specified as well automatic design features related to the goal of a particular study and the intended experimental setting. In particular, we demonstrate that our method effectively designs PCR and sequencing primers for the 454 system.

## Availability and requirements

The software is available as a web application at http://www.hiv.lanl.gov/tools/primer/main. As a web tool, it is platform independent. Like other bioinformatic tools at the HIV database, the tool will be maintained in the long term, and users can suggest new features as well as report problems to seq-info@lanl.gov. Running it as a web tool therefore also guarantees that a user always will use the latest version.

**Project name:** HIV databases; PrimerDesign

**Project home page:**http://www.hiv.lanl.gov/tools/primer/main

**Operating system(s):** Platform independent

**Programming language:** Perl, C, Catalyst, Moose

**Other requirements:** web browser

**License:** Copyright 2012. Los Alamos National Security, LLC. This material was produced under U.S. Government contract DE-AC52-06NA25396 for Los Alamos National Laboratory (LANL), which is operated by Los Alamos National Security, LLC for the U.S. Department of Energy. The U.S. Government has rights to use, reproduce, and distribute this software. NEITHER THE GOVERNMENT NOR LOS ALAMOS NATIONAL SECURITY, LLC MAKES ANY WARRANTY, EXPRESS OR IMPLIED, OR ASSUMES ANY LIABILITY FOR THE USE OF THIS SOFTWARE. If software is modified to produce derivative works, such modified software should be clearly marked, so as not to confuse it with the version available from LANL. Additionally, this program is free software; you can redistribute it and/or modify it under the terms of the GNU General Public License as published by the Free Software Foundation; version 2.0 of the License. Accordingly, this program is distributed in the hope that it will be useful, but WITHOUT ANY WARRANTY; without even the implied warranty of MERCHANTABILITY or FITNESS FOR A PARTICULAR PURPOSE. See the GNU General Public License for more details.

**Any restrictions to use by non-academics:** see license

## Competing interests

The authors declare that they have no competing interests.

## Authors’ contributions

Wrote code: JB, MK, GA; Performed DNA sequencing: CG, LG; Designed experiments: TL, BK, WF, PH, LG; Wrote paper: TL, JB, MK, BK, WF, GA, PH, LG, CG. All authors read and approved the final manuscript.
